# A Japanese man with malaria tests negative for fever after spending 6 months in rural Kenya

**DOI:** 10.1186/s41182-020-00220-z

**Published:** 2020-06-24

**Authors:** Takashi Sugimoto, Kensuke Takahashi, Kosuke Matsui, Masahito Asada, Osamu Kaneko, Koya Ariyoshi

**Affiliations:** 1 Department of Infectious Diseases, Nagasaki University Hospital, Nagasaki University, Nagasaki, Japan; 2 Department of Clinical Medicine, Institute of Tropical Medicine, Nagasaki University, Nagasaki, Japan; 3 Department of Protozoology, Institute of Tropical Medicine, Nagasaki University, Nagasaki, Japan

## Abstract

A previously healthy Japanese man in his fifties was admitted to our hospital because of a recurrent fever after returning from Kenya and Madagascar. He was ambulant with a body temperature of 36.6 °C. His physical examination revealed normal except for tender hepatomegaly. The blood test results showed no apparent abnormality except thrombocytopenia and mild liver dysfunction. The rapid diagnostic test and Giemsa-stained blood film were repeatedly negative for malaria. Computed tomography scans of the chest, abdomen, and pelvis revealed no significantly abnormal findings.

## History

A previously healthy Japanese man in his fifties was admitted to the Department of Infectious Disease at Nagasaki University Hospital in Japan because of a fever after returning from a 6-month stay in Kenya. The patient stayed in Madagascar from November 20YY-1 to January 20YY prior to his stay a rural city in Kenya from January to late June 20YY to conduct behavioral research at a community hospital. According to the patient, the hospital ward consisted of many febrile patients, including malaria patients, but his research work did not involve direct contact with any patients. He also reported having many mosquito bites and walking on muddy roads since it was the rainy season. He drank tea with unpasteurized milk from water buffalo for breakfast, ate local food for lunch, and cooked supper for himself. He drank bottled water throughout the stay. He was vaccinated against hepatitis A and B, tetanus, rabies, typhoid fever, and yellow fever prior to his travel, but he had not taken anti-malarial chemoprophylaxis. He denied having sexual contact.

Twenty days (day x-8) after returning to Japan, he experienced episodes of fever with headache, fatigue, and back pain. Five days before admission, his body temperature increased to more than 38 °C, and he had frequent urination. On the following day, he visited a local clinic where he was prescribed levofloxacin and acetaminophen and diagnosed with urinary tract infection. Two days before admission, his temperature increased to 39.6 °C at night and was accompanied by chills; thus, he visited our outpatient clinic on the next day (day x-1).

## Clinical examination

Upon examination at the first visit, he was ambulant with a body temperature of 36.6 °C, a blood pressure of 108/85 mmHg, a pulse of 85 bpm, and a respiratory rate of 14 per minute. His abdominal examination revealed tender hepatomegaly. There was no rash, no lymphadenopathy, no bleeding tendency, and no joint pain. The remainder of the physical examination results was normal. The blood test results showed no apparent abnormality except moderate thrombocytopenia and mild liver dysfunction: WBC 6500/μL (segment 74.6%, lymphocyte 18.9%), hemoglobin 14.0 g/dL, platelets 7.4 × 10^4^/μL, total bilirubin 0.8 mg/dL, AST 42 U/L, ALT 50 U/L, BUN 13 mg/dL, and creatinine 0.77 mg/dL. The urinalysis showed proteinuria (±), occult blood in the urine (−), and WBC (−). The rapid diagnostic test (BinaxNOW®) and Giemsa-stained blood film were negative for malaria. Computed tomography (CT) scans of the chest, abdomen, and pelvis revealed no significantly abnormal findings.

**Fig. 1:**
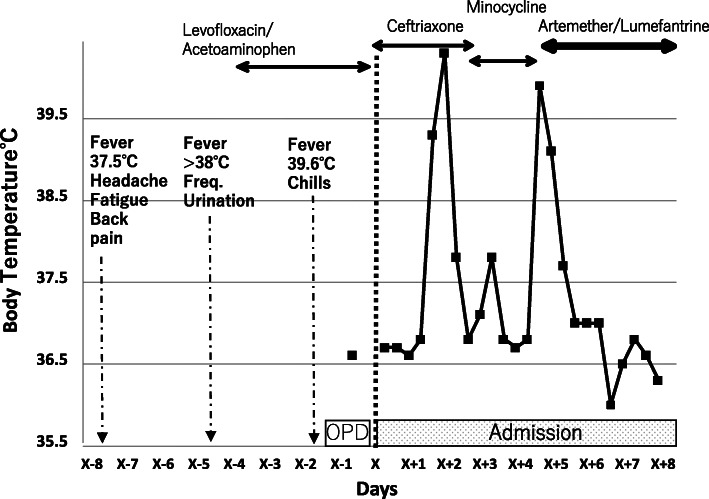
Clinical course and body temperature

On day x, the patient was admitted for further investigation. Intravenous ceftriaxone 2 g per day was initiated after two sets of blood cultures were taken. On the second hospital day (day x+1), the rapid diagnostic test and Giemsa-stained blood test were repeated, and the results were negative. However, his body temperature increased again at night. On the third hospital day, the blood culture results were negative. Ceftriaxone was discontinued, and oral minocycline 100 mg every 12 h was started. On the 5th hospital day (day x+4), his body temperature increased again at night (Fig. 1). Additional diagnostic tests were ordered.

## Clinical questions

1. What are the important differential diagnoses?
2. Which diagnostic tests should be performed?

## Diagnosis and clinical course

We requested to conduct a Brucella agglutination reaction test at an external commercial laboratory; an IgM immunofluorescent assay (IFA) and PCR assays for rickettsia and Borrelia at the national reference laboratory, the National Institute of Infectious Diseases in Tokyo; and PCR assays for malaria at the Institute of Tropical Medicine at Nagasaki University.

On the 6th hospital day (day x+5), it was reported that PCR amplification targeting malaria *cytb* was positive, and the sequence analysis showed that it matched with *Plasmodium malariae*. The Giemsa-stained films of the blood samples were re-examined by an experienced malariologist, and they revealed a low level of malaria parasites with the proportion of erythrocytes ranging from < 0.006 to 0.018%. Thus, the patient was diagnosed with quartan malaria and treated with artemether/lumefantrine for 3 days. On the 9th hospital day, defervescence was achieved, and the patient was discharged. The results of the Brucella agglutination reaction (*B. abortus* and *B. canis*) were negative. Anti-rickettsia antibodies (*Orientia* species and *Rickettsia japonica*) and PCR assays for rickettsia and Borrelia species were not performed eventually because malaria was diagnosed, and the fever did not respond to minocycline. One and a half years after discharge, the patient remained free from recrudescence.

## Discussion

In evaluating febrile patients who travelled to malaria-endemic countries, it is imperative to pay special attention to exclude falciparum malaria, as it can rapidly progress and cause death unless the patient is promptly treated with anti-malaria drugs. Therefore, if there is any clinical suspicion of malaria, malaria tests should be repeated at least three times with 12- to 24-h interval to exclude malaria [1].

In the current case study, malaria was the number one suspected disease among the probable differentials, and malaria tests, including the rapid diagnosis test and blood smear examination, were repeated and showed negative results. It is worth knowing that in the setting of travel medicine, sensitivity of rapid diagnosis tests for *P. falciparum* is significantly lower (median 74.1%) if the parasitemia level was below 100/μL compared with above 100/μL (median 94.3%) [2] and that in cases of *Plasmodium malariae* infection, the parasitemia level remains very low; thus, the presence of parasitemia is often missed by malaria blood film tests [3, 4]. Furthermore, the malaria rapid diagnosis test used in the current case is based on an immunochromatographic method with detection antibodies targeting both *P. falciparum*-specific histidine-rich-protein-2 (PfHRP2) and a pan-malarial antigen, *Plasmodium* aldolase, but the sensitivity of this test is lower for non-falciparum malaria, especially in the detection of *P. ovale* and *P. malariae*, probably due to the lower affinity of some monoclonal antibodies to these species [5]. PCR assays, on the other hand, are capable of identifying malaria with both high sensitivity and high specificity. A disadvantage of PCR is that it is not rapid or a point of care in routine clinical practice. However, PCR needs to be conducted to diagnose conditions such as *P. malariae* infection, as the current case exemplifies. Would the patient have been treated empirically with antimalarial drugs if PCR assays had not been available? The answer highly depends on other factors, such as clinical severity, access to antimalarial drugs, and in this case, we would less likely have started empirical malaria treatment without knowledge of *P. malariae* disease.

He also had moderate thrombocytopenia, but we considered it rather non-specific reaction and did not investigate further, because his general condition was good, and DIC was excluded. It returned to normal rapidly following malaria treatment.

Treating a fever of an individual who has returned from traveling abroad, especially in tropical countries, is challenging for physicians working in high-income countries because (a) they have to diagnose tropical infectious diseases, for which clinicians often have little experience; (b) some tropical infectious diseases, such as falciparum malaria, are fatal; and (c) other diseases, such as typhoid fever, lead to significant public health threats to the local population. In approaching the fever of a returned traveller, thorough and directional evaluations are essential: comprehensive history taking, examinations to differentiate critical and curable diseases, and timely consultations with experts should be conducted when necessary. Collecting the latest epidemiological information of endemic diseases is also crucial. The prevalence of *P. malariae* is much lower than that of *P. falciparum* but not uncommon. According to recent reports from Kenya [6] and Madagascar [7], the prevalence of *P*. *malariae* among symptomatic patients was 3.3% and 1.2%, respectively, while that of *P*. *falciparum* was 77% and 52%, respectively. Moreover, the vast majority of *P*. *malariae* infections were coinfected with *P*. *falciparum*. Therefore, *P*. *malariae* infection is often overlooked and neglected in the local population.

Finally, the fever pattern should also be carefully observed. It was noted that the patient in this case study had a fever that was periodic with an exact 72-h interval, which could have been a clue for the diagnosis of quartan malaria (Fig. 1).

## Final diagnosis

Quartan malaria (*P. malariae* infection)

## Data Availability

All data generated or analyzed during this study are included in this published article.

## Abbreviations

WBC: White blood cell
AST: Aspartate transaminase
ALT: Alanine transaminase
BUN: Blood urea nitrogen
CT: Computed tomography
IFA: Immunofluorescent assay
PCR: Polymerase chain reaction
PfHRP1: *P*. *falciparum*-specific histidine-rich-protein-2
